# Optical Spectroscopy and Crystal Field Analysis of Ni^2+^ Centres in Lithium Tetraborate Glass

**DOI:** 10.3390/ma19204303

**Published:** 2026-10-13

**Authors:** Ihor Kindrat, Bohdan Padlyak, Adam Drzewiecki, Radosław Lisiecki

**Affiliations:** 1 Institute of Physics, University of Zielona Góra, 4a Szafrana Str., 65-516 Zielona Góra, Poland; 2 O.G. Vlokh Institute of Physical Optics, Ivan Franko National University of Lviv, 23 Dragomanov Str., 79005 Lviv, Ukraine; 3 Division of Optical Spectroscopy, Institute of Low Temperature and Structure Research of the Polish Academy of Sciences, 2 Okólna Str., 50-422 Wrocław, Poland

**Keywords:** borate glass, Ni^2+^ ions, optical absorption, luminescence, Tanabe–Sugano diagram

## Abstract

The Ni-doped Li_2_B_4_O_7_ (Li_2_O–2B_2_O_3_) glass was prepared by the conventional melt-quenching method. Structural and spectroscopic properties of the glass were investigated using X-ray diffraction (XRD), optical absorption, and photoluminescence spectroscopy, including excitation, emission, and decay kinetics. The absence of sharp diffraction peaks in the XRD pattern confirms the disordered glassy structure of the investigated glass. The optical absorption spectrum shows three broad bands associated with spin-allowed transitions of Ni^2+^ (3*d*^8^) ions. The excitation and emission spectra of the Li_2_B_4_O_7_:Ni glass reveal several spin-allowed and spin-forbidden 3*d*–3*d* transitions of Ni^2+^ ions. In particular, the Li_2_B_4_O_7_:Ni glass exhibits broad green and red emissions centred at approximately 525 and 687 nm, respectively, as well as weak near-infrared emission bands in the 900–1100 and 1500–1750 nm regions. The analysis of the crystal field splitting for Ni^2+^ ions in the Li_2_B_4_O_7_:Ni glass is carried out, and the Racah parameters (*B* and *C*) and the crystal field strength (*Dq*) are calculated. The Tanabe–Sugano diagram for the octahedrally coordinated Ni^2+^ ions in the Li_2_B_4_O_7_:Ni glass is built and discussed.

## 1. Introduction

Borate glasses are glassy materials that contain boron oxide (glass former) and various other oxides (glass modifiers). These glasses have interesting optical properties and are used in various applications such as solid-state lighting, laser materials, scintillation detectors, and optical fibres [1,2,3,4]. The preparation of borate glasses is simple and cost-effective compared with complicated methods for growing the corresponding single crystals. Besides this, the properties of borate glasses can be easily modified by doping with rare-earth and/or transition metal impurities. The presence of nickel (Ni) impurity in borate glasses is useful for many technological applications [5,6,7]. In particular, the Ni-doped borate glasses can be used as (1) luminescent materials for solid-state lighting; (2) the active medium in optical fibre amplifiers; (3) the gain medium in laser systems; (4) the transparent layers in photovoltaic devices, such as solar cells, to enhance the efficiency of light absorption.

This work is focused on studies of Ni-doped borate glass with the Li_2_B_4_O_7_ (Li_2_O–2B_2_O_3_) chemical composition, which is equivalent to the well-known lithium tetraborate crystal [8,9,10]. The Li_2_B_4_O_7_:Ni glass can be easily fabricated into a variety of shapes and sizes, which is practically impossible for the crystalline analogue. The spectroscopic terms of transition metal ions, including nickel ions, are split in the crystal field according to the ligand field theory, depending on the symmetry of the local environment of 3*d* ions. It should be noted that a full understanding of the optical and structural properties of Ni-doped Li_2_B_4_O_7_ glass is necessary to exploit its potential for various optical and electronic applications.

In this work, we use X-ray diffraction (XRD) and optical spectroscopy (absorption and luminescence) techniques to investigate the spectroscopic properties of the Ni-doped Li_2_B_4_O_7_ glass. XRD is a powerful technique for the structural characterisation of a material. In this work, XRD was used to confirm the disordered glassy structure of the investigated material. Optical absorption and luminescence spectroscopy was used to obtain valuable information about the electronic and local structure of nickel ions.

The Ni impurity is typically incorporated into the network of oxide glasses as Ni^2+^ (3*d*^8^) ions, which can be detected by optical absorption spectroscopy due to the presence of three intense spin-allowed absorption bands in the visible to near-infrared (Vis–NIR) spectral range. The optical spectra of Ni^2+^ ions are dependent on their local surroundings. The four-, five-, and/or six-coordinated Ni^2+^ ions can be observed in glasses depending on the glass composition and the content and nature of modifiers. In particular, Ni^2+^ ions in the tetrahedral geometry (four-fold coordination) have been observed in potassium silicate glasses [11]. The Ni^2+^ ions in the trigonal bipyramidal geometry (five-fold coordination) have been observed in calcium and magnesium silicate and various borosilicate glasses [11,12]. The Ni^2+^ ions in the octahedral geometry (six-fold coordination) are dominant in alkali borate glasses [12,13].

The position of the optical absorption bands of Ni^2+^ ions is dependent on the crystal field strength, which decreases with lowering of coordination number. As a consequence, the optical absorption bands of Ni^2+^ in the tetrahedral configuration are considerably red-shifted compared to those in the octahedral configuration. It has been summarised in [14] that the colour of a glass sample can indicate the coordination number of the oxygen anions around the Ni^2+^ cation. Glasses with Ni^2+^ ions in the four-fold coordination reveal a blue colour because the most intense absorption band is located in the red spectral range [14]. Glasses containing Ni^2+^ ions in the five-fold coordination have an intense brown colour because a very broad absorption band is located in the blue-green spectral range [14]. The most intense absorption band of Ni^2+^ ions in the six-fold coordination is located in the blue spectral region, resulting in a yellow colour for the corresponding glasses [14]. Therefore, the positions and shapes of the Ni^2+^ absorption bands provide valuable information about the coordination geometry and degree of structural distortion surrounding the impurity ion.

Several studies have reported the spectroscopic properties of Ni-doped borate glasses with chemical compositions similar to the investigated glass. For instance, the optical absorption of Ni-doped borate glasses with 20R_2_O–80B_2_O_3_ composition (where R = Li, Na and K) was studied in [15]. The absorption spectrum of the Ni-doped Li_2_O–4B_2_O_3_ glass showed a band at 416 nm, which was assigned to the ^3^A_2g_ (F)–^3^T_1g_ (P) transition of Ni^2+^ ions in the octahedral coordination [15]. However, article [15] did not examine the optical absorption in the NIR spectral region, perform luminescence studies, and analyse crystal field parameters. Another study [16] reported the optical absorption of Ni-doped sodium zinc borate glass with 20Na_2_O–15ZnO–64B_2_O_3_–1NiO composition. The observed two bands with maxima at 424 nm and 799 nm were assigned to spin-allowed transitions of Ni^2+^ ions in the octahedral field [16]. In bismuth borate glass with 29Bi_2_O_3_–70B_2_O_3_–1NiO composition, an absorption band at 423 nm was observed, which was due to the spin-allowed ^3^A_2g_ (F) → ^3^T_1g_ (P) transition of Ni^2+^ ions in the octahedral geometry [17]. However, analysis of the crystal field splitting of Ni^2+^ ions is missing in [17]. Three broad absorption bands at 425, 793, and 1264 nm assigned to the ^3^A_2g_(^3^F) → ^3^T_1g_(^3^P), ^3^T_lg_(^3^F), and ^3^T_2g_(^3^F) spin-allowed transitions of Ni^2+^ ions in the octahedral coordination were observed in the lead borate glass with 49.5PbO–50B_2_O_3_–0.5NiO chemical composition [18]. However, study [18] did not analyse the crystal field or perform detailed luminescence studies.

The available referenced data indicate that the luminescent properties and crystal field splitting in Ni-doped borate glasses have not been thoroughly studied. In particular, a comprehensive spectroscopic description of the Ni-doped Li_2_B_4_O_7_ glass is still lacking. Accordingly, the present work aims to provide a comprehensive structural and spectroscopic characterisation of the Li_2_B_4_O_7_:Ni glass. XRD was employed to examine the glassy structure of the investigated material. UV–Vis–NIR absorption and photoluminescence spectroscopy were used to identify the oxidation state and the electronic transitions of Ni impurity ions. The luminescence decay kinetics were analysed to assess the influence of multiple radiative channels and structural disorder on the excited-state relaxation. Finally, the experimental transition energies were used to determine the crystal-field strength and Racah parameters and to construct a Tanabe–Sugano diagram. The combination of the obtained results provides valuable insight into the electron structure, ligand crystal-field environment, and spectroscopic behaviour of the Ni impurity centres in the Li_2_B_4_O_7_:Ni glass.

## 2. Materials and Methods

### 2.1. Preparation of the Ni-Doped Li_2_B_4_O_7_ Glass

The Ni-doped lithium tetraborate glass with the nominal composition Li_2_B_4_O_7_ (or Li_2_O–2B_2_O_3_) was prepared by the conventional melt-quenching method under the technological conditions described in [19]. The starting materials used for the preparation of the Li_2_B_4_O_7_ host composition were lithium carbonate (Li_2_CO_3_, purity—≥99.9%, Sigma-Aldrich) and boric acid (H_3_BO_3_, purity—99.97%, Sigma-Aldrich), in amounts of 0.1 and 0.4 mol, respectively. The reagents were weighed in stoichiometric proportions, thoroughly mixed and loaded into a 60 mL corundum crucible. The solid-state synthesis of the Li_2_B_4_O_7_ compound was carried out using the following multi-step chemical reaction:

(1) $$\begin{array}{c} {\mathrm{Li}}_{2}{\mathrm{CO}}_{3}{+4H}_{3}{\mathrm{BO}}_{3}\overset{{4H}_{2}O↑}{\underset{170{}^{\circ}C}{\to }}{\mathrm{Li}}_{2}{\mathrm{CO}}_{3}+4(\alpha \text{-}{\mathrm{HBO}}_{2})\overset{{2H}_{2}O↑}{\underset{250{}^{\circ}C}{\to }}\\ {\mathrm{Li}}_{2}{\mathrm{CO}}_{3}{+2B}_{2}O_{3}\overset{{\mathrm{CO}}_{2}↑}{\underset{600{}^{\circ}C}{\to }}x{\mathrm{Li}}_{2}B_{4}O_{7}+(1-x)\left[{\mathrm{Li}}_{2}{\mathrm{CO}}_{3}+2B_{2}O_{3}\right]\overset{{\mathrm{CO}}_{2}↑}{\underset{800{}^{\circ}C}{\to }}{\mathrm{Li}}_{2}B_{4}O_{7} \end{array}$$

The nickel impurity was introduced in the form of nickel (II) oxide (NiO, purity—99.99%, Sigma-Aldrich) at a nominal concentration of 1.0 mol.%. The Li_2_B_4_O_7_:Ni glass was obtained by a quick cooling of the corresponding melt. To avoid the crystallisation processes, the melt was heated approximately 100 K above the melting point of Li_2_B_4_O_7_ (T_melt_ = 917 °C, or 1190 K) [19]. The process of glass preparation was carried out in the air. The resulting glass samples were cut and polished to sizes of approximately 10 × 6 × 2 mm^3^ for optical absorption and photoluminescence measurements. Meanwhile, small pieces were used for the XRD studies.

### 2.2. Experimental Techniques

The XRD studies were carried out with usage of a computer-controlled X-ray diffractometer (model DRON-3, NPO “Burevestnik”, Leningrad, USSR) with Cu Kα radiation source. The XRD diffractogram was obtained in the 2*θ* angle range of 5–145° with a scanning speed of 2.0°/min. The optical absorption spectrum of the Li_2_B_4_O_7_:Ni glass was measured using the Agilent Cary 5000 UV–Vis–NIR spectrophotometer (Agilent Technologies, Inc., Santa Clara, CA, USA). The spectrophotometer includes a double-beam optical system, deuterium and tungsten halogen lamps, a double-grating monochromator, an R928 photomultiplier tube, and a cooled PbS detector.

The luminescence emission and excitation spectra in the UV–Vis spectral range, as well as slow decay kinetics, were registered using the Horiba FluoroMax–4 spectrofluorimeter (HORIBA, Ltd., Kyoto, Japan). The spectrofluorimeter includes a 150 W xenon lamp, excitation and emission grating monochromators, and an R928P photomultiplier tube. The luminescence emission spectra in the NIR spectral region were registered using the Dongwoo Optron DM711 monochromator (Dongwoo Optron Co., Ltd., Gwangju-si, Republic of Korea) and an InGaAs detector (Hamamatsu, Japan). The NIR luminescence was excited using a high-power LED at λ_exc_ = 445 nm. The fast decay kinetics was registered using an experimental setup containing a Continuum Surelite optical parametric oscillator (OPO) pumped by the third harmonic of a pulsed Nd:YAG laser, a GDM 1000 grating monochromator (Carl Zeiss Jena, Jena, Germany), a Hamamatsu R955 photomultiplier tube (Hamamatsu Photonics K.K., Hamamatsu City, Japan), and a Tektronix MSO44 oscilloscope (Tektronix, Inc., Beaverton, OR, USA). All investigations were performed at room temperature.

## 3. Results and Discussion

### 3.1. XRD Characterisation

**Figure 1:**
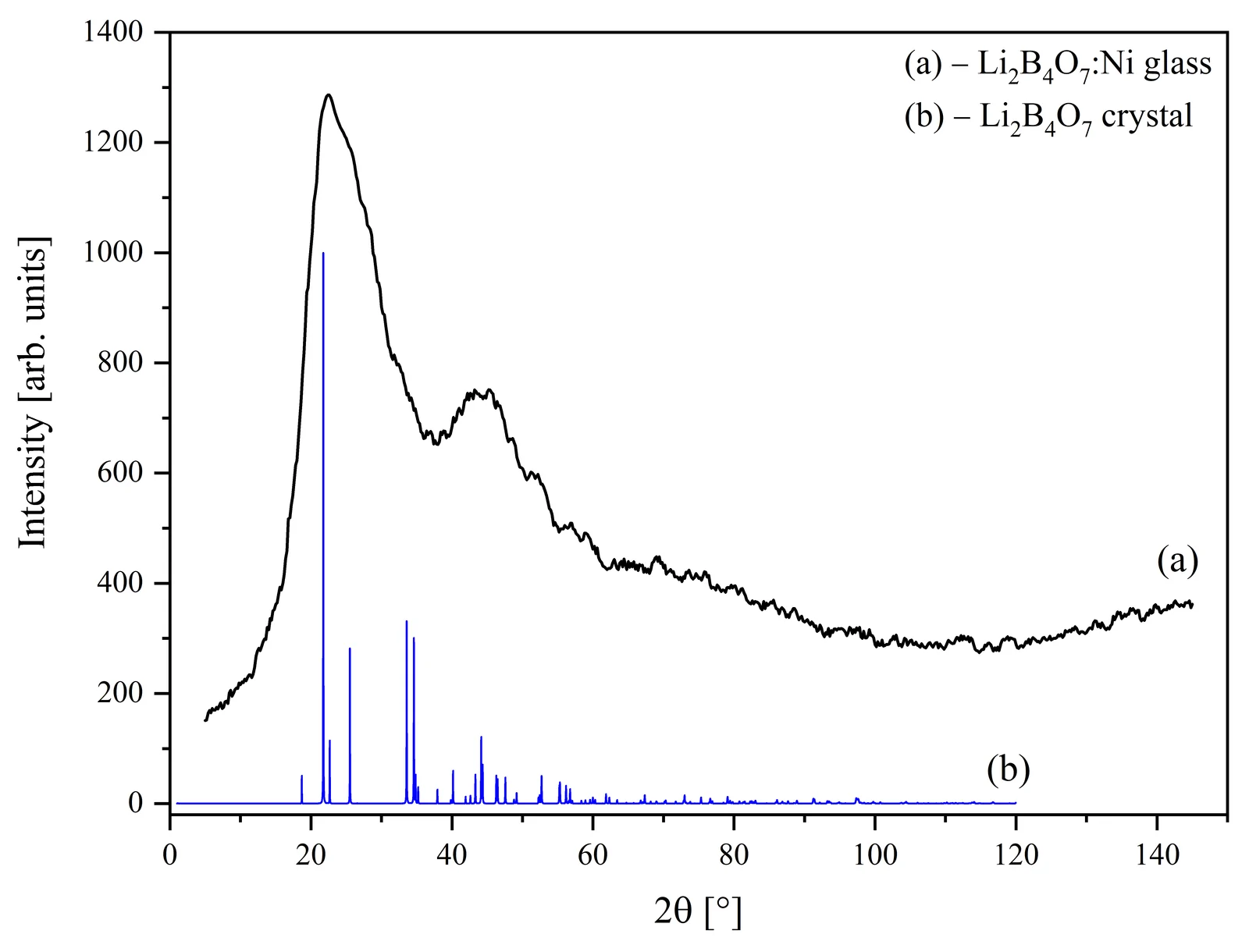
The experimental XRD profile of the Li_2_B_4_O_7_:Ni glass (black curves (a)) and the diffraction peaks of the Li_2_B_4_O_7_ crystal (blue curves (b)).

Figure 1 shows the experimental X-ray diffraction pattern of the Li_2_B_4_O_7_:Ni glass together with the reference diffraction profile of crystalline Li_2_B_4_O_7_ calculated from the crystallographic data reported in [20]. The experimental XRD pattern of the Li_2_B_4_O_7_:Ni glass is characterised by a broad diffuse scattering halo and the absence of sharp Bragg reflections, indicating the absence of detectable long-range crystalline order and confirming the disordered glassy (vitreous) structure of the investigated sample. The position of the main diffuse maximum is consistent with the region of the strongest reflections of crystalline Li_2_B_4_O_7_, suggesting that the short-range structural properties of the glass retain some similarity to those of the crystalline phase.

### 3.2. Optical Absorption and Crystal-Field Analysis

**Figure 2:**
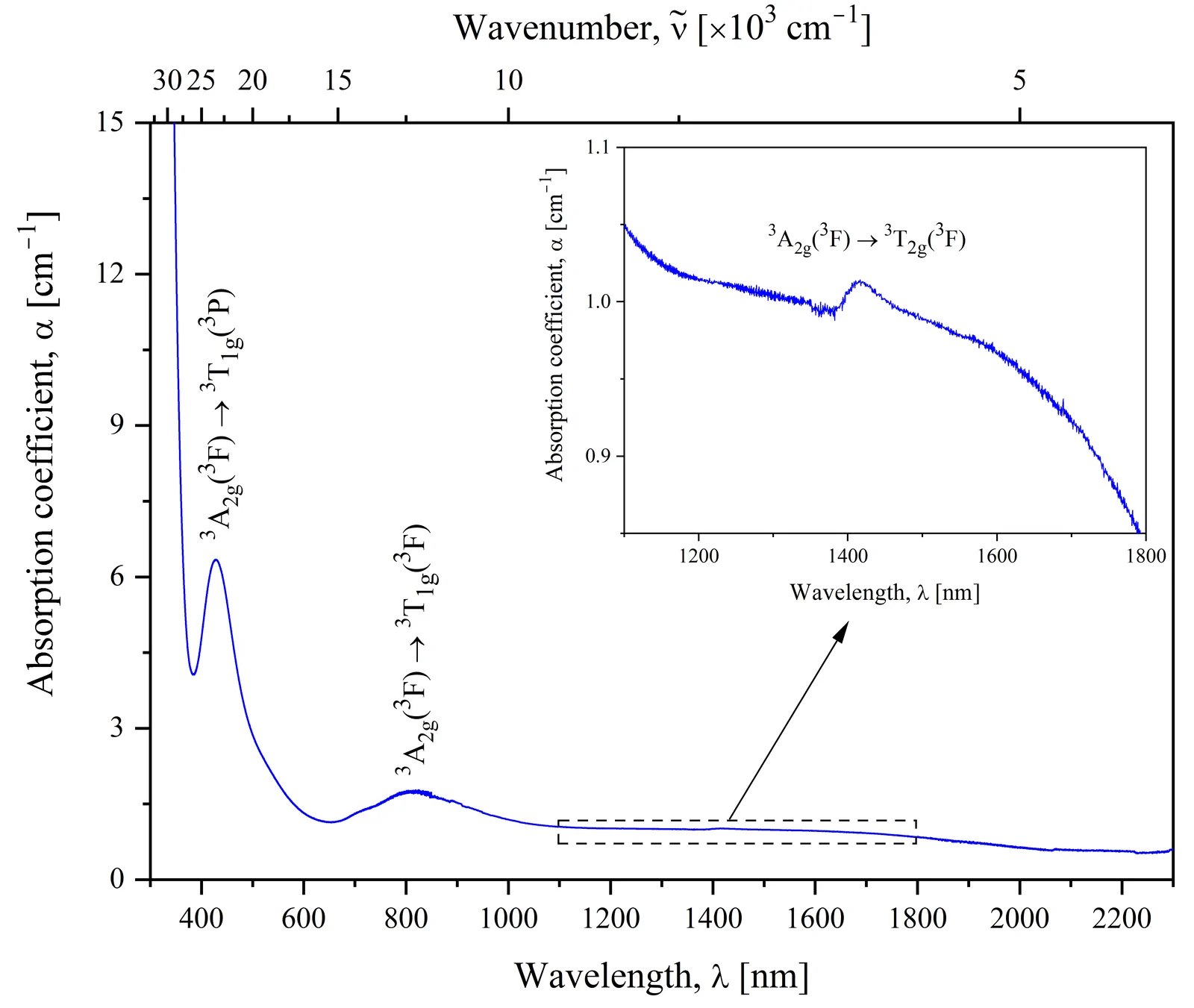
Optical absorption spectrum of the Li_2_B_4_O_7_:Ni glass registered in the UV–Vis–NIR spectral range.

The UV–Vis–NIR optical absorption spectrum of the Li_2_B_4_O_7_:Ni glass is shown in Figure 2. The fundamental absorption edge of the glass host is manifested by a steep increase in the absorbance approximately between 370 nm and 320 nm. In addition to the host absorption edge, three broad absorption bands are observed at 428 nm (23,364 cm^−1^), 815 nm (12,270 cm^−1^), and 1417 nm (7057 cm^−1^). The absorption band at 428 nm is the strongest, whereas the absorption band at 1417 nm is the weakest. After careful examination and verification, the observed absorption bands are related to the 3*d*–3*d* transitions of Ni^2+^ ions.

The Ni^2+^ ions contain 8 electrons in the unfilled 3*d* shell and have five *LS* terms: two spin-triplets (^3^F and ^3^P) and three spin-singlets (^1^D, ^1^G, and ^1^S). The relative positions of the spectroscopic terms can be expressed in terms of the Racah parameters *B* and *C* [21]. The ^2S+1^L terms are split under the influence of the crystal field. In particular, in the octahedral crystal field the ^3^F ground term splits into the ^3^A_2g_, ^3^T_2g_, and ^3^T_1g_ states, the ^3^P term transforms to the ^3^T_1g_ state, the ^1^D term splits into the ^1^E_g_ and ^1^T_2g_ states, the ^1^G term splits into the ^1^A_1g_, ^1^T_1g_, ^1^E_g_, and ^1^T_2g_ states, and the ^1^S term transforms to the ^1^A_1g_ state. The crystal field splitting can be analysed using the Tanabe–Sugano diagram [22], which depicts the dependence of *E*/*B* on *Dq*/*B* for a fixed *C*/*B* ratio, where *E* is the energy of the crystal field state and *Dq* is the strength of the crystal field.

The observed three broad absorption bands peaking at 428 nm, 815 nm, and 1417 nm are assigned to the spin-allowed transitions ^3^A_2g_ (^3^F) → ^3^T_2g_ (^3^F), ^3^A_2g_ (^3^F) → ^3^T_1g_ (^3^F), and ^3^A_2g_ (^3^F) → ^3^T_1g_ (^3^P), respectively. The relatively large bandwidths of these transitions are consistent with the distribution of local crystal-field environments expected for Ni^2+^ centres in a structurally disordered glass. Spin-forbidden triplet-singlet transitions are not observed in Figure 2 due to their low intensity caused by the spin-forbidden character. Besides this, the inhomogeneous broadening of spin-allowed absorption bands caused by the disordered structure of the Li_2_B_4_O_7_:Ni glass can hide weak triplet-singlet transitions.

The energies of the three triplet crystal field states can be expressed analytically as functions of the crystal-field parameter *Dq* and the Racah parameter *B* by solving the corresponding Tanabe–Sugano matrices. The explicit expressions used in the present analysis are the following:

(2) *E*(^3^*T*_2_*_g_*(^3^*F*)) = 10*Dq*

(3) $$E{(}^{3}T_{1g}{(}^{3}F))=15\mathrm{Dq}+7.5B-0.5\sqrt{100Dq^{2}-180\mathrm{DqB}+225B^{2}}$$

(4) $$E{(}^{3}T_{1g}{(}^{3}P))=15\mathrm{Dq}+7.5B+0.5\sqrt{100Dq^{2}-180\mathrm{DqB}+225B^{2}}$$

Using the energies obtained from the optical absorption spectrum, it is possible to calculate the *Dq* and *B* values from different pairs of transitions. It is possible to obtain three sets of *Dq* and *B* values using Equations (2) and (3), Equations (2) and (4), and Equations (3) and (4). As a result, slightly different values of the *Dq* and *B* parameters were obtained depending on the equations used for the calculation. In particular, *Dq* = 706 cm^−1^ was obtained using Equations (2) and (3), and Equations (2) and (4), *B* = 943 cm^−1^ was obtained using Equations (2) and (4) as well as *Dq* = 727 cm^−1^ and *B* = 921 cm^−1^ were obtained using Equations (3) and (4). The differences between the obtained values are expected for a structurally disordered glass host, where the local crystal field is not perfectly described in the framework of an ideal octahedral model.

A weak shoulder near 520 nm is visible on the long-wavelength side of the strongest absorption band at 428 nm. This shoulder can be associated with Ni^2+^ ions in the trigonal bipyramidal geometry (five-fold coordination) according to [11,12]. However, this assignment cannot be considered unambiguous because the shoulder lies close to the intense 428 nm absorption band and may also contain contributions from weak spin-forbidden transitions or from the splitting and broadening of crystal-field states in a distorted octahedral environment. Moreover, additional absorption bands that could be unambiguously assigned to the five-coordinated Ni^2+^ ions were not observed or were hidden by the broad absorption of the six-coordinated Ni^2+^ ions. On the one hand, the attribution of the shoulder around 520 nm to the five-coordinated Ni^2+^ ions has sense, but on the other hand, it can also be associated with several inhomogeneously broadened spin-forbidden transitions of Ni^2+^ ions or with the distortion of the octahedral field (Jahn-Teller effect). Therefore, in the further analysis only the three well-defined absorption maxima related exclusively to the octahedral geometry of the ligand field are considered.

To obtain a single consistent set of crystal-field parameters, the three experimental transition energies were fitted simultaneously using a least-squares procedure. The *Dq* and *B* parameters were determined by minimising the root mean square deviation between the experimental energies obtained from the optical absorption spectrum and the theoretical energies calculated from Equations (2)–(4). Using this method, it is possible to obtain *Dq* = 720.3 cm^−1^ and *B* = 928.7 cm^−1^. Consequently, the *Dq*/*B* ratio equals 0.776 and indicates a weak crystal field in the Li_2_B_4_O_7_:Ni glass. A strong crystal field condition usually corresponds to the relation *Dq*/*B* > 1.6, in which the first excited crystal field state is ^1^E_g_ (^1^D) instead of ^3^T_2g_ (^3^F) for the weak crystal field.

It should be noted that the theoretical energies given by Equations (2)–(4) are non-linearly dependent on the *Dq* and *B* parameters. As a result, obtaining uncertainties of the *Dq* and *B* values is a very complicated task. Therefore, the Monte Carlo method according to the procedure described in [23] was used to estimate the uncertainties of the fitted parameters. Two hundred sets of simulated transition energies were generated by randomly varying the experimental energies within their respective uncertainties. For each simulated data set, *Dq* and *B* were recalculated using the same least-squares fitting procedure as for the experimental data. The resulting distributions of *Dq* and *B* were approximately Gaussian, with standard deviations of 7.3 and 4.9 cm^−1^, respectively. The 95% confidence intervals give *Dq* = 720 ± 15 cm^−1^ and *B* = 929 ± 10 cm^−1^. The obtained *Dq*/*B* = 0.776 ± 0.024 value in the Li_2_B_4_O_7_:Ni glass will be used below for the identification of Ni^2+^ transitions in the luminescence spectra using the Tanabe–Sugano diagram [22].

### 3.3. Luminescence Excitation and Emission Spectra in the UV–Vis Range

**Figure 3:**
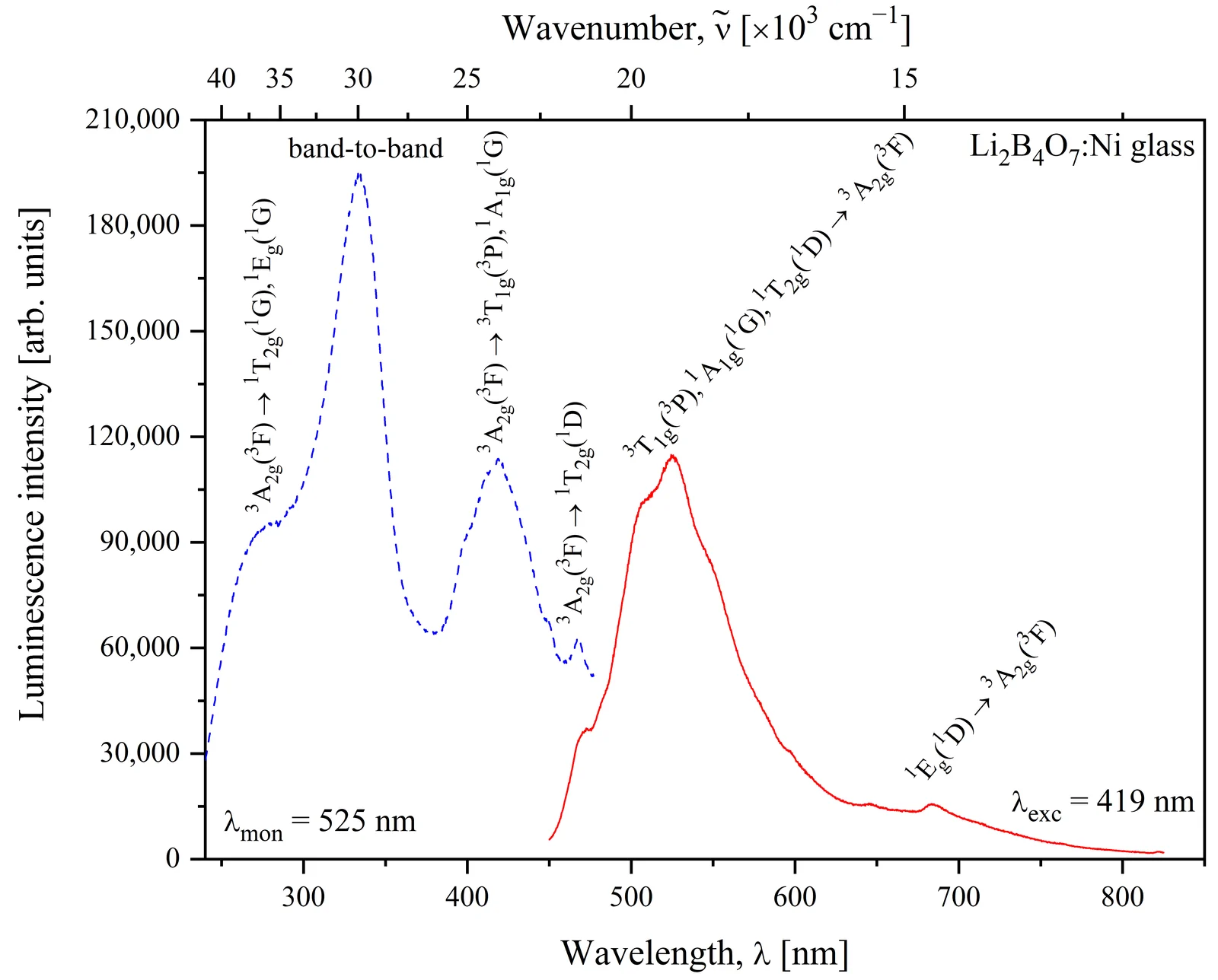
Luminescence excitation (blue dashed curve) and emission (red solid curve) spectra of the Li_2_B_4_O_7_:Ni glass, registered under excitation at *λ*_exc_ = 419 nm and by monitoring at *λ*_mon_ = 525 nm, respectively.

The strongest Ni^2+^ absorption band is at 428 nm; therefore, excitation in the violet-blue spectral region is particularly effective for investigating the luminescence of the Ni-doped Li_2_B_4_O_7_ glass. The photoluminescence excitation and emission spectra of the Li_2_B_4_O_7_:Ni glass are presented in Figure 3. First, a series of emission spectra was registered under excitation at different wavelengths in the violet-blue spectral region in order to determine the excitation wavelength providing the highest emission intensity. The emission spectrum of the Li_2_B_4_O_7_:Ni glass was registered upon excitation at 419 nm and is given by the red curve in Figure 3.

The emission spectrum exhibits a strong broad band at 525 nm (19,048 cm^−1^) and a much weaker band near 684 nm (14,620 cm^−1^). The full width at half maximum (FWHM) of the green emission band equals 77 nm (2787 cm^−1^). The emission band peaked at 525 nm belongs to the ^3^T_1g_ (^3^P) → ^3^A_2g_ (^3^F) transition of Ni^2+^ ions. It should be noted that the ^1^A_1g_ (^1^G) → ^3^A_2g_ (^3^F) and ^1^T_2g_ (^1^D) → ^3^A_2g_ (^3^F) spin-forbidden transitions can also contribute to the green emission of the Li_2_B_4_O_7_:Ni glass, as the ^1^A_1g_ (^1^G) and ^1^T_2g_ (^1^D) states lie in the vicinity of the ^3^T_1g_ (^3^P) state at *Dq*/*B* = 0.776. The weak emission band peaked near 684 nm belongs to the ^1^E_g_ (^1^D) → ^3^A_2g_ (^3^F) transition of Ni^2+^ ions.

The excitation spectrum of the Li_2_B_4_O_7_:Ni glass given in Figure 3 was registered by monitoring the intensity of the emission peak at 525 nm. The excitation spectrum consists of an intense band at 419 nm, a weak band at 467 nm, a very intense band at 334 nm, and a shoulder near 280–290 nm. The intense band at 419 nm (23,866 cm^−1^) belongs to the ^3^A_2g_ (^3^F) → ^3^T_1g_ (^3^P) transition of Ni^2+^ ions. The ^3^A_2g_ (^3^F) → ^1^A_1g_ (^1^G) transition can also contribute to the band at 419 nm because the ^3^T_1g_ (^3^P) and ^1^A_1g_ (^1^G) states are very close at *Dq*/*B* = 0.776. The weak band at 467 nm (21,413 cm^−1^) is related to the ^3^A_2g_ (^3^F) → ^1^T_2g_ (^1^D) transition of Ni^2+^ ions. The very intense band at 334 nm cannot be related to nickel impurity, because Ni^2+^ ions in the Li_2_B_4_O_7_:Ni glass do not exhibit any spin-allowed transitions in the UV region within the present crystal-field model. The band at 334 nm agrees well with the fundamental absorption edge of the glass host and can be attributed to the band-to-band transition of the Li_2_B_4_O_7_:Ni glass. The shoulder near 280–290 nm can be assigned to the ^3^A_2g_ (^3^F) → ^1^E_g_ (^1^G) and ^3^A_2g_ (^3^F) → ^1^T_2g_ (^1^G) transitions of Ni^2+^ ions.

**Figure 4:**
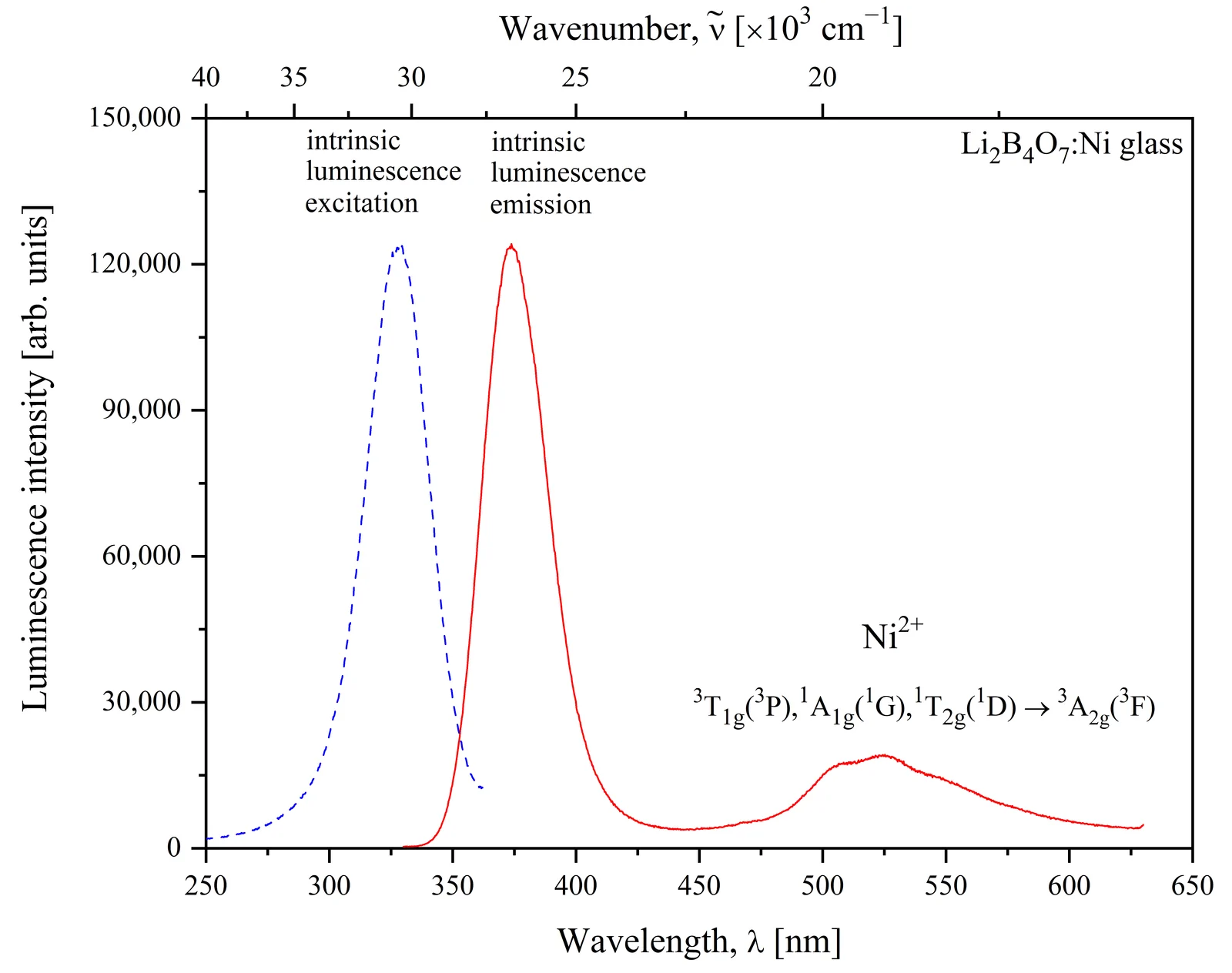
Luminescence excitation (blue dashed curve) and emission (red solid curve) spectra associated with the intrinsic luminescence in the Li_2_B_4_O_7_:Ni glass.

The emission and excitation spectra of the intrinsic luminescence caused by the band-to-band electron-hole recombination are given in Figure 4. The results obtained for the Li_2_B_4_O_7_:Ni glass are very similar to those reported by us for undoped borate glasses in [24]. In particular, the undoped borate glasses with Li_2_B_4_O_7_, LiKB_4_O_7_, CaB_4_O_7_, and LiCaBO_3_ compositions exhibit a luminescence band in the 360–400 nm spectral region with very fast decay kinetics upon excitation at 310–340 nm [24]. The emission spectrum of the Li_2_B_4_O_7_:Ni glass given in Figure 4 shows the intrinsic host-related emission band at 374 nm and the Ni^2+^ emission band at 525 nm. The presence of Ni^2+^ luminescence under excitation in the host absorption region is consistent with energy transfer from the glass network to the nickel impurity.

**Figure 5:**
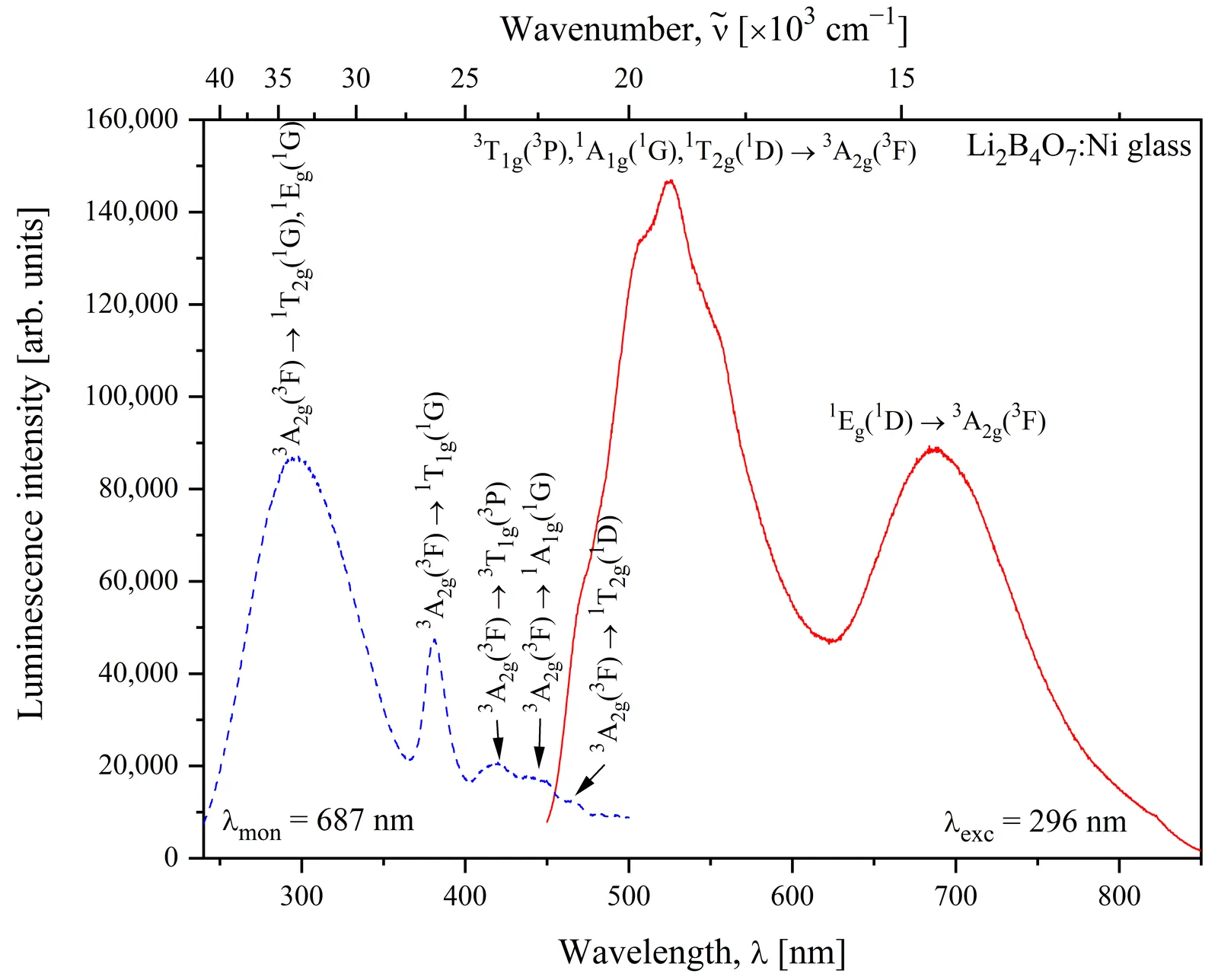
Luminescence excitation (blue dashed curve) and emission (red solid curve) spectra of the Li_2_B_4_O_7_:Ni glass, registered under excitation at *λ*_exc_ = 296 nm and by monitoring *λ*_mon_ = 687 nm, respectively.

In addition to the green luminescence, the Li_2_B_4_O_7_:Ni glass also exhibits a weaker red emission. The excitation spectrum of the ^1^E_g_ (^1^D) → ^3^A_2g_ (^3^F) emission band of Ni^2+^ ions in the Li_2_B_4_O_7_:Ni glass is shown in Figure 5. It can be seen that the excitation spectrum of the red emission in Figure 5 is different from the excitation spectrum of the green emission given in Figure 3. The excitation spectrum given in Figure 5 consists of an intense broad band at 296 nm, an intense band at 381 nm, and three weak bands in the spectral range of 405–475 nm. The intense broad band with a maximum at 296 nm (33,784 cm^−1^) and FWHM of 78 nm (8665 cm^−1^) corresponds to the ^3^A_2g_ (^3^F) → ^1^E_g_ (^1^G) and ^3^A_2g_ (^3^F) → ^1^T_2g_ (^1^G) transitions of Ni^2+^ ions. The broad nature of this band can also be caused by the influence of the band-to-band transition of the glass host. The band peaked at 381 nm (26,247 cm^−1^) with FWHM of 22 nm (1547 cm^−1^) belongs to the ^3^A_2g_ (^3^F) → ^1^T_1g_ (^1^G) transition of Ni^2+^ ions. Finally, the three weak bands in the spectral range of 405–475 nm belong to the ^3^A_2g_ (^3^F) → ^3^T_1g_ (^3^P), ^3^A_2g_ (^3^F) → ^1^A_1g_ (^1^G), and ^3^A_2g_ (^3^F) → ^1^T_2g_ (^1^D) transitions of Ni^2+^ ions. The relative enhancement of the red emission under UV excitation compared with violet-blue excitation is consistent with the increased contribution of higher-lying singlet states to the excitation pathways of the red emission.

**Figure 6:**
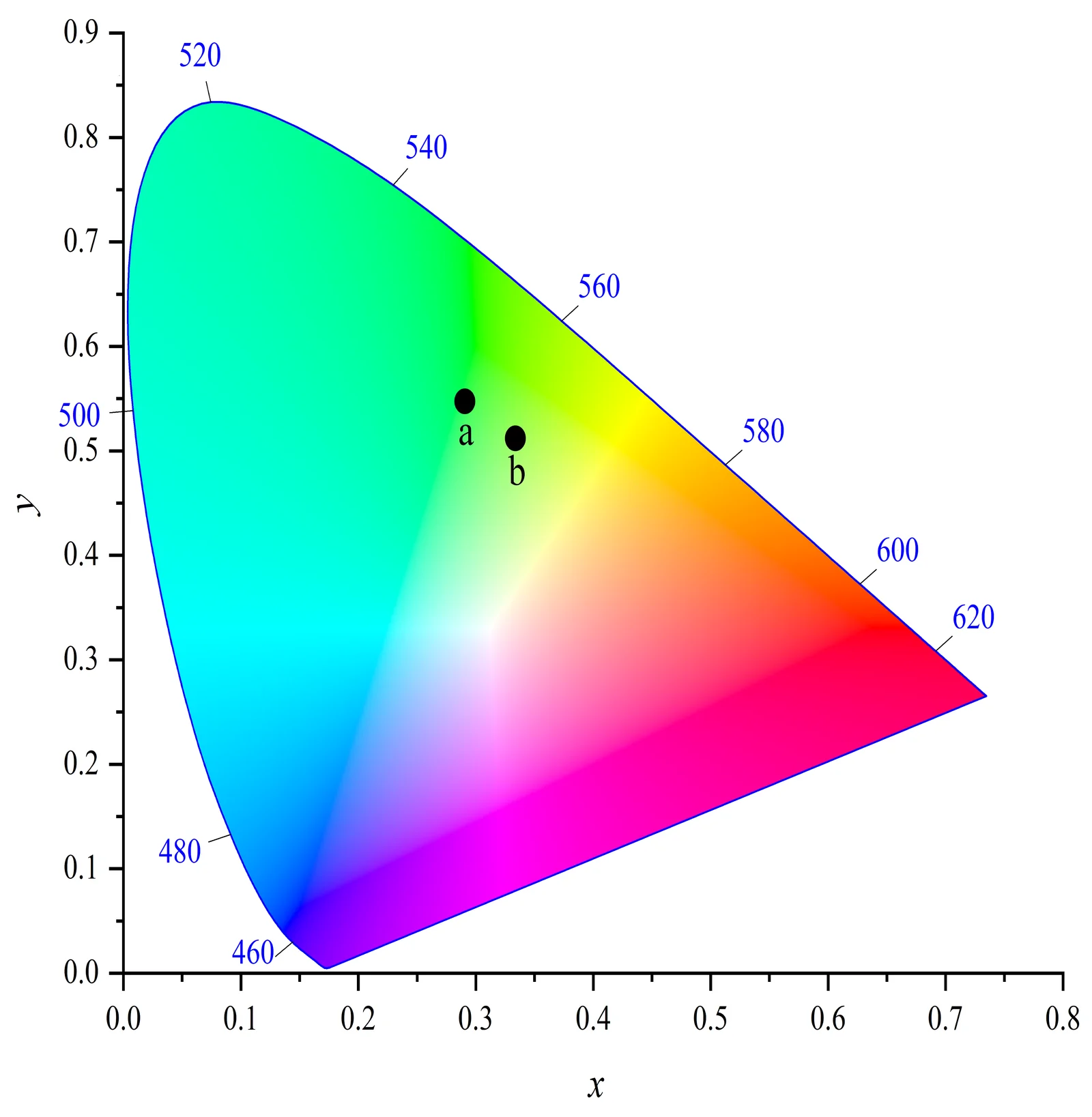
CIE diagram showing the emission colour coordinates of the Li_2_B_4_O_7_:Ni glass upon excitation at 419 nm (marked with “•” and labelled as (a)) and 296 nm (marked with “•” and labelled as (b)).

The CIE chromaticity diagram of the Li_2_B_4_O_7_:Ni glass is given in Figure 6. The chromaticity coordinates were determined using the CIE 1931 standard colourimetric system [25] with the 2° standard observer. First, the CIE tristimulus values (X, Y, and Z) were calculated by integrating the spectral data multiplied by the colour-matching functions. Then, the chromaticity coordinates (*x*, *y*) were calculated from these tristimulus values. Further details of the calculation procedure can be found elsewhere [26]. The emission colour of the investigated glass under excitation at 419 nm and 296 nm is represented by the symbol “•” marked by (a) and (b), respectively. It can be seen that the emission colour of the Li_2_B_4_O_7_:Ni glass under the UV and violet-blue excitation is quite similar. Only a slight shift from the green to the yellowish-green spectral region can be observed due to the increase in intensity of the red Ni^2+^ emission under UV excitation.

### 3.4. Luminescence Emission Spectra in the NIR Range

**Figure 7:**
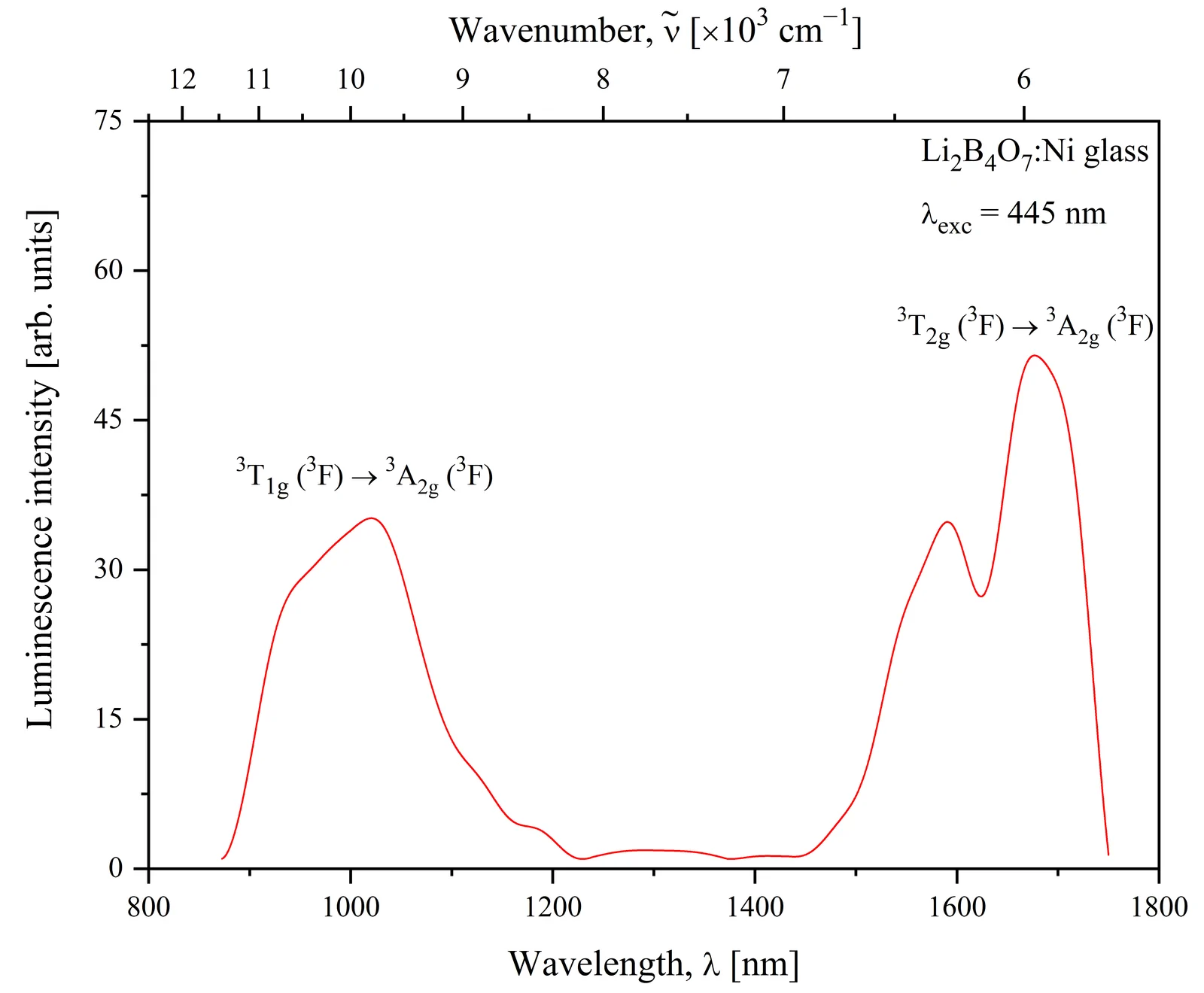
Luminescence emission spectrum of the Li_2_B_4_O_7_:Ni glass, registered in the NIR spectral range under excitation at *λ*_exc_ = 445 nm using an LED source.

The NIR emission spectrum of the Li_2_B_4_O_7_:Ni glass registered upon excitation at 445 nm using an LED source is shown in Figure 7. The spectrum consists of two bands in the spectral ranges of 900–1100 nm and 1500–1750 nm. The first band, with a peak at 1020 nm and a shoulder near 940 nm, can be ascribed to the spin-allowed ^3^T_1g_ (^3^F) → ^3^A_2g_ (^3^F) transition. The second band with a main peak at 1677 nm and an additional peak at 1591 nm can be ascribed to the spin-allowed ^3^T_2g_ (^3^F) → ^3^A_2g_ (^3^F) transition.

Considering the shapes of the absorption and emission bands, one can observe varying degrees of band splitting, which is particularly pronounced for some bands, while others show only weak evidence of this feature. Two main mechanisms may account for this splitting: (1) distortion of the octahedral coordination environment (O_h_ symmetry) to shortened and/or elongated octahedra with lower D_4h_ symmetry, and (2) spin–orbit coupling of double (E) and triple (T) degenerate states. Among the observed absorption and emission bands, the NIR emission bands exhibit the most pronounced deviations from a simple Gaussian profile. In our opinion, the shape of the NIR emission bands is associated with distortion of the octahedral coordination environment rather than with spin–orbit splitting. In particular, for the shortened and/or elongated octahedra, the ^3^A_2g_ (^3^F) ground state transforms to the ^3^B_1g_ state, the ^3^T_2g_ (^3^F) state splits into the ^3^E_g_ and ^3^B_2g_ states, and the ^3^T_1g_ (^3^F) state splits into the ^3^A_2g_ and ^3^E_g_ states. Thus, the considered splitting of the ^3^T_2g_ (^3^F) and ^3^T_1g_ (^3^F) states into two tetragonal states well describes the shape of the NIR emission bands consisting of two spectral components. In the case of spin–orbit splitting, three components are expected for the triply degenerate ^3^T_2g_ (^3^F) and ^3^T_1g_ (^3^F) crystal field states, which do not correlate well with the doublet structure of the observed NIR emission bands. Nevertheless, the contribution of spin–orbit splitting in the Li_2_B_4_O_7_:Ni glass cannot be entirely excluded, taking into account the relatively large spin–orbit coupling constant of Ni^2+^ ions and the possibility that inhomogeneous broadening may obscure the individual spin–orbit components. It is quite possible that the electronic states initially split by the lowering of symmetry may undergo additional splitting due to spin–orbit coupling. Similar to observed NIR emission bands, the splitting or asymmetry observed in some of the other absorption and emission bands may also arise primarily from distortions of the octahedral coordination environment, although contributions from spin–orbit coupling may also be present.

The intensity of the observed luminescence in the NIR spectral region is significantly weaker than that in the visible spectral region. However, a quantitative comparison is very complicated because the luminescence shown in Figure 3, Figure 5 and Figure 7 was registered using different instruments with different detector types. The weaker intensity of the NIR emission can be attributed to the increasing of non-radiative processes caused by multiphonon relaxation.

Near-infrared emission from Ni^2+^ centres in glasses has been reported only in a limited number of studies. An intense Ni^2+^ emission near 1300 nm was observed in glass ceramics containing LiGa_5_O_8_:Ni^2+^ nanocrystals [27]. The broadband near-infrared emission band with a peak at 1400 nm and a bandwidth exceeding 300 nm attributed to the ^3^T_2g_ (^3^F) → ^3^A_2g_ (^3^F) transition of Ni^2+^ ions was registered in Ni-doped zinc–alumino–silicate glass by the scientists from Japan in [28]. The crystal field strength in the ZnO–Al_2_O_3_–SiO_2_ glass was *Dq* = 932 cm^−1^ [28]. This value is higher than the *Dq* in the Li_2_B_4_O_7_:Ni glass, and consequently the corresponding emission band in [28] has a higher energy in comparison with our result. It was concluded in [28] that Ni-doped glass has the potential to serve as an amplification medium for broadband optical amplifiers and tuneable lasers. In our opinion, further studies are needed in order to evaluate the potential of Ni-doped Li_2_B_4_O_7_ glass for use in the field of optoelectronics.

### 3.5. Luminescence Decay Kinetics

**Figure 8:**
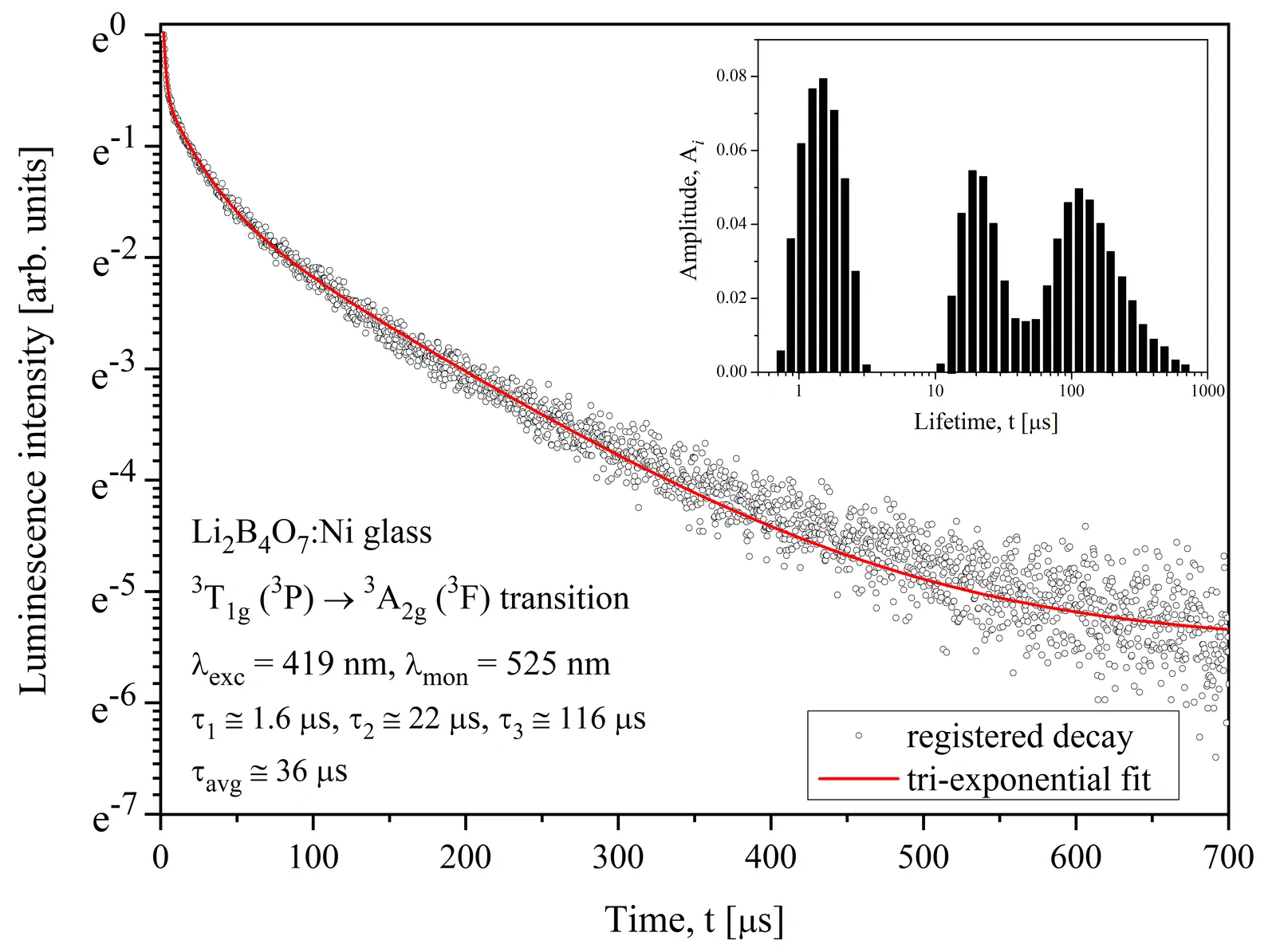
Luminescence decay kinetics of the green Ni^2+^ emission in the Li_2_B_4_O_7_:Ni glass. The experimental decay curve and tri-exponential fit are shown together with the corresponding distribution of luminescence lifetimes in the inset.

The luminescence decay kinetics of the green Ni^2+^ emission with a maximum at 525 nm in the Li_2_B_4_O_7_:Ni glass is shown in Figure 8. The decay curve exhibits a pronounced non-exponential character and cannot be adequately described by a single-exponential function. A bi-exponential fit also gives an unsatisfactory description of the experimental data. But, the decay curve is well fitted by a tri-exponential function with lifetimes of 1.6 μs, 22 μs, and 116 μs. The weighted amplitudes (also called fractional or relative amplitudes) of this tri-exponential fit are 0.432, 0.327, and 0.241, respectively. The amplitude-weighted average lifetime (*τ*_avg_) was calculated using the following formula:

(5) $$\tau_{\mathrm{avg}}=\frac{\sum_{i}A_{i}\tau_{i}}{\sum_{i}A_{i}},$$

where *A_i_* is the amplitude of the *τ_i_* lifetime. The evaluated *τ*_avg_ lifetime equals 36 μs.

The pronounced non-exponential decay behaviour of the green emission band may be attributed to several factors: (1) contributions from different Ni^2+^ electronic transitions with different radiative and non-radiative relaxation rates; (2) local symmetry lowering, which lifts the degeneracy of Ni^2+^ electronic states and may lead to their splitting; (3) the distribution of local coordination environments in the glass network. In our opinion, all three decay components of the tri-exponential fit are primarily associated with the spin-allowed ^3^T_1g_ (^3^P) → ^3^A_2g_ (^3^F) transition. The nearly spectrally located spin-forbidden ^1^A_1g_ (^1^G) → ^3^A_2g_ (^3^F) and ^1^T_2g_ (^1^D) → ^3^A_2g_ (^3^F) transitions may affect the measured decay kinetics, causing the lifetime of the ^3^T_1g_ (^3^P) → ^3^A_2g_ (^3^F) transition to be longer. Nevertheless, it is not possible to unambiguously assign individual components of the tri-exponential fit to these spin-forbidden transitions. Regarding the effect of local symmetry lowering, distortion of the ideal octahedral coordination environment through shortening or elongation may reduce the symmetry from O_h_ to D_4h_. This distortion can lift the degeneracy of the ^3^T_1g_ (^3^P) emitting state, resulting in two tetragonal states, ^3^A_2g_ and ^3^E_g_. Their potentially different relaxation rates may give slightly different decay components. In addition, variations in Ni–O bond lengths and local crystal-field strength in the distorted octahedra can produce a distribution of relaxation rates and, consequently, a distribution of lifetimes.

**Figure 9:**
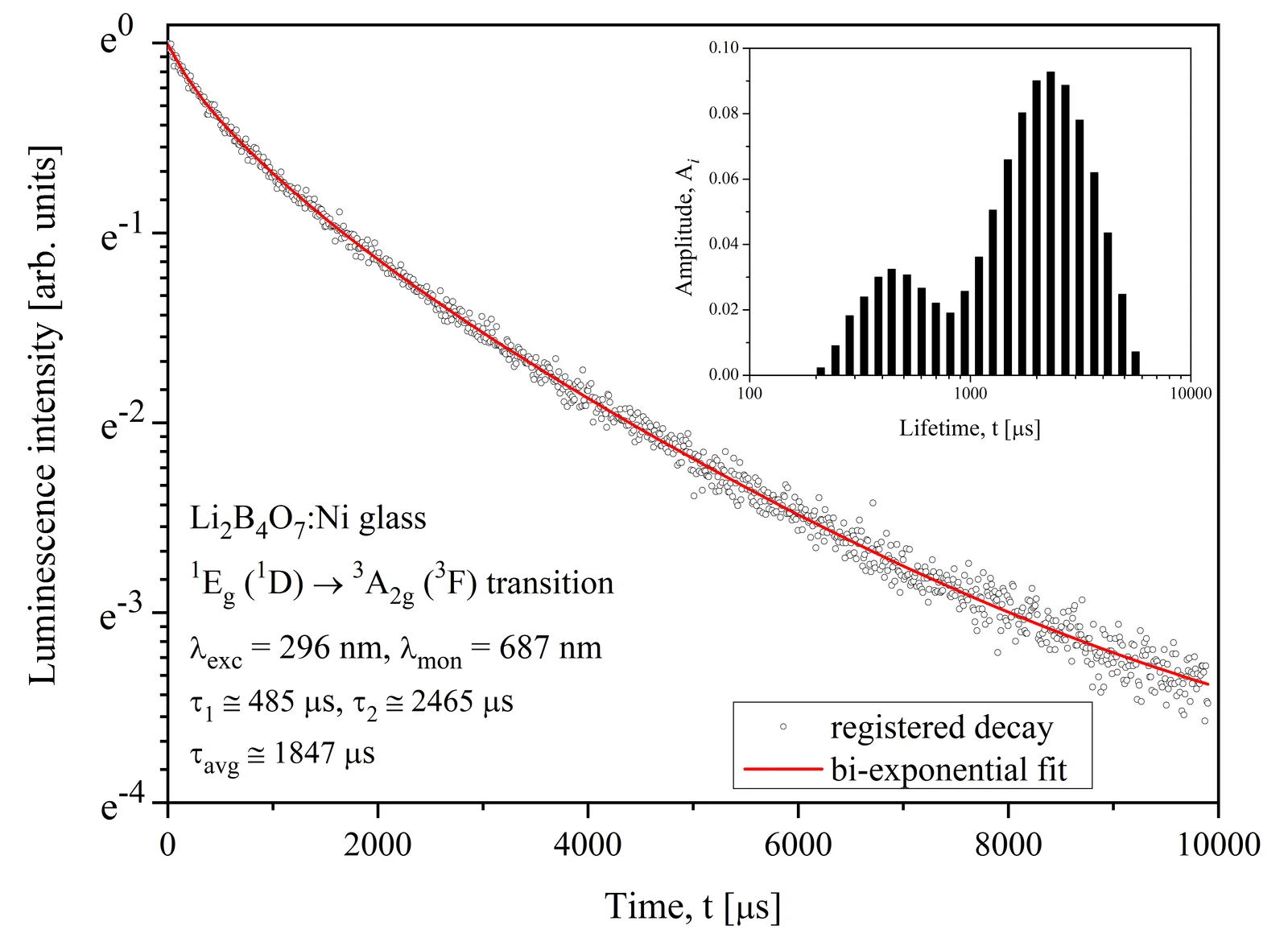
Luminescence decay kinetics of the red Ni^2+^ emission in the Li_2_B_4_O_7_:Ni glass. The experimental decay curve and bi-exponential fit are shown together with the corresponding distribution of luminescence lifetimes in the inset.

Figure 9 shows the luminescence decay kinetics of the red Ni^2+^ emission peaked at 687 nm in the Li_2_B_4_O_7_:Ni glass. The decay curve is also non-single exponential, but its deviation from single-exponential behaviour is considerably weaker than that observed for the green Ni^2+^ emission. The decay curve was well fitted by a bi-exponential function with lifetimes of 485 μs and 2465 μs. The weighted amplitudes of this bi-exponential fit are 0.312 and 0.688, respectively. The amplitude-weighted average lifetime calculated using the Formula (5) equals 1847 μs. The red Ni^2+^ emission is assigned to the spin-forbidden ^1^E_g_ (^1^D) → ^3^A_2g_ (^3^F) transition. Its decay is more than 50 times slower than that of the green emission, which is dominated by the spin-allowed ^3^T_1g_ (^3^P) → ^3^A_2g_ (^3^F) transition.

Since there are no other crystal field states located near the ^1^E_g_ (^1^D) state at *Dq*/*B* = 0.776 in the Li_2_B_4_O_7_:Ni glass, the contribution of other electronic transitions to the kinetics of the red Ni^2+^ emission is insignificant. The non-exponential decay behaviour of the red emission may be associated with local symmetry lowering and the distribution of local coordination environments in the glass network. In a tetragonally distorted octahedral coordination environment, the ^1^E_g_ (^1^D) emitting state splits into the ^1^A_1g_ and ^1^B_1g_ states. Transitions from these tetragonal states may exhibit slightly different relaxation rates, potentially giving two decay components with slightly different lifetimes. In addition, variations in bond lengths and local crystal-field strength in the distorted octahedra formed by Ni^2+^ ions and O^2−^ ligands can lead to a distribution of relaxation rates and, consequently, to a distribution of observed lifetimes.

The luminescence lifetimes of the NIR emission bands in the Li_2_B_4_O_7_:Ni glass were found to be in the microsecond range, but the low emission intensity of the NIR luminescence did not allow registration of decay curves of good quality.

To obtain additional information on the distribution of decay kinetics, a continuous lifetime-distribution analysis was performed. It was assumed that the total emission is the result of a superposition of luminescence from a considerable number of Ni^2+^ luminescence centres, each localised in sites with slightly different local surroundings. Besides this, the continuous distribution of the local crystal field in the Li_2_B_4_O_7_:Ni glass causes a continuous distribution of the lifetimes. The calculated emission intensity can be expressed as follows [29]:

(6) $$I(t)=\int \frac{A(\tau )}{\tau }e^{-\frac{t}{\tau }}\mathrm{d\tau}=\int A(\tau )e^{-\frac{t}{\tau }}d(\mathrm{ln\tau}),$$

where *A*(*τ*) is the distribution function of lifetime *τ*. The continuous lifetime distribution was approximated by a discrete set of logarithmically spaced lifetimes in order to go from integration to summation and to facilitate the calculation. The amplitudes *A_i_*(*τ_i_*) associated with logarithmically spaced lifetimes were determined by fitting the calculated decay curve to the experimental data using a least-squares procedure.

The lifetime distributions obtained for the green and red Ni^2+^ emission are given as insets in Figure 8 and Figure 9, respectively. The obtained distributions support the appropriateness of the tri- and bi-exponential fitting models for the green and red Ni^2+^ emissions, respectively. The maxima of the distributions correlate very well with the corresponding fitted lifetimes. From the obtained lifetime distribution of the green Ni^2+^ emission, it can be seen that the luminescence lifetimes are distributed in the time range of 0.7–700 μs. Such wide lifetime distribution is caused by three effects: first, the green emission band contains contributions from both spin-allowed and spin-forbidden Ni^2+^ transitions with different relaxation rates; second, local symmetry lowering causes splitting of Ni^2+^ octahedral states into tetragonal states with potentially slightly different relaxation rates; third, structural disorder of the glass produces a distribution of local crystal-field environments and, consequently, a distribution of Ni^2+^ relaxation rates. For the red emission, the lifetime distribution extends approximately from 200 to 6000 μs and is substantially narrower than that obtained for the green emission. Since the red emission is caused by the single spin-forbidden ^1^E_g_ (^1^D) → ^3^A_2g_ (^3^F) transition, this distribution is attributed to the local symmetry lowering and positional disorder of the glass network, which produces a distribution of Ni^2+^ relaxation rates.

### 3.6. Tanabe–Sugano Diagram of Ni^2+^ Ions in the Li_2_B_4_O_7_:Ni Glass

The Tanabe–Sugano diagram describes the dependence of the state energies on the crystal-field strength as the relationship between *E*/*B* and *Dq*/*B* for a fixed *C*/*B* ratio. For the Li_2_B_4_O_7_:Ni glass, the analysis of the optical absorption spectrum give *Dq* = 720 ± 15 cm^−1^, *B* = 929 ± 10 cm^−1^, and *Dq*/*B* = 0.776 ± 0.024. In order to build the Tanabe–Sugano diagram, the *C* parameter and the *C*/*B* ratio must also be determined. The *C* parameter cannot be calculated from the positions of the spin-allowed transitions used to obtain *Dq* and *B*, because their energies are independent of *C* (see Equations (2)–(4)). To find the *C* parameter, the energy of a spin-forbidden transition must be used. Obtaining the energy from the emission spectrum is less reliable because the emission energy can be affected by the Stokes shift between absorption and emission. Consequently, obtaining the energy from the excitation spectrum is a better option. Analysis of the excitation spectra given in Figure 3 and Figure 5 suggests that the ^3^A_2g_ (^3^F) → ^1^T_1g_ (^1^G) transition is the best candidate for calculation of the *C* parameter. The corresponding band with a maximum at 381 ± 1 nm (26,247 ± 69 cm^−1^) has a relatively high intensity and does not overlap with other identified Ni^2+^ transitions. From the Tanabe–Sugano theory, the theoretical energy of the ^1^T_1g_ (^1^G) state can be analytically determined to be equal to:

(7) *E*(^1^*T*_1_*_g_*(^1^*G*)) = 10*Dq* + 12*B* + 2*C*.

As a result, the Racah parameter *C* and the *C*/*B* ratio in the Li_2_B_4_O_7_:Ni glass equal 3950 ± 170 cm^−1^ and 4.25 ± 0.23, respectively. The uncertainties were calculated using the law of uncertainty propagation.

It is important to compare the obtained Racah parameters *B* = 929 ± 10 cm^−1^ and *C* = 3950 ± 170 cm^−1^ with the corresponding values *B*_0_ and *C*_0_ for free Ni^2+^ ions. According to reference [30], free Ni^2+^ ions exhibit *B*_0_ = 1030 cm^−1^ and *C*_0_ = 4850 cm^−1^. The *B*/*B*_0_ and *C*/*C*_0_ ratios equal 0.9019 ± 0.0097 and 0.814 ± 0.035, respectively, and indicate a reduction in the interelectronic repulsion parameters in the glass relative to the free Ni^2+^ ion. The obtained *β* = *B*/*B*_0_ ratio is typical for oxygen ligands in the context of the nephelauxetic effect. It should be noted that in [31] slightly different values *B*_0_ = 1068 cm^−1^ and *C*_0_ = 4457 cm^−1^ for free Ni^2+^ ions were reported. In this case, the ratios *B*/*B*_0_ = 0.8699 ± 0.0094 and *C*/*C*_0_ = 0.886 ± 0.038 are very similar to each other. Although the absolute ratios depend on the selected free-ion reference values, both sets of parameters indicate a reduction in the Racah parameters relative to the free-ion case.

**Figure 10:**
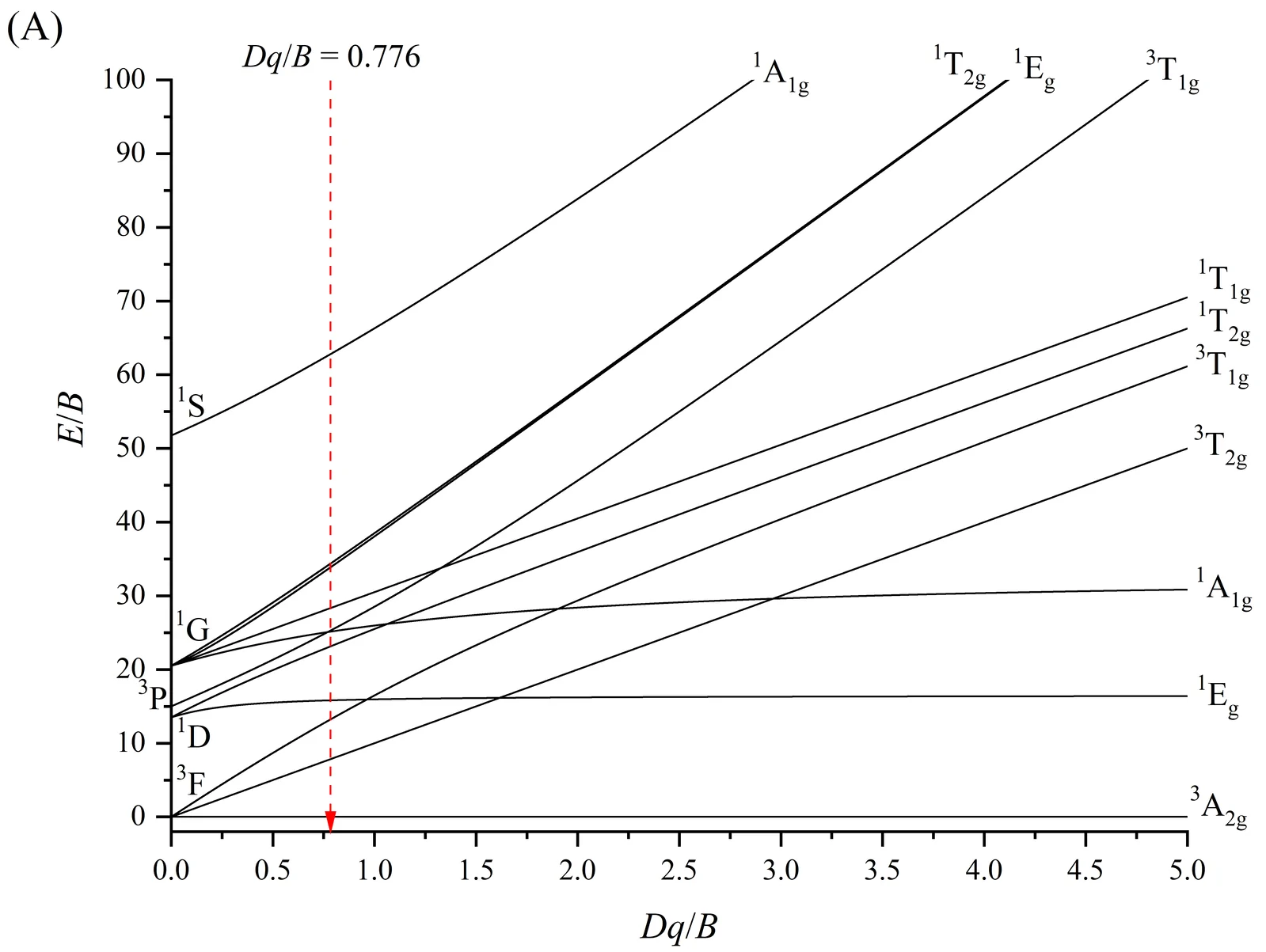

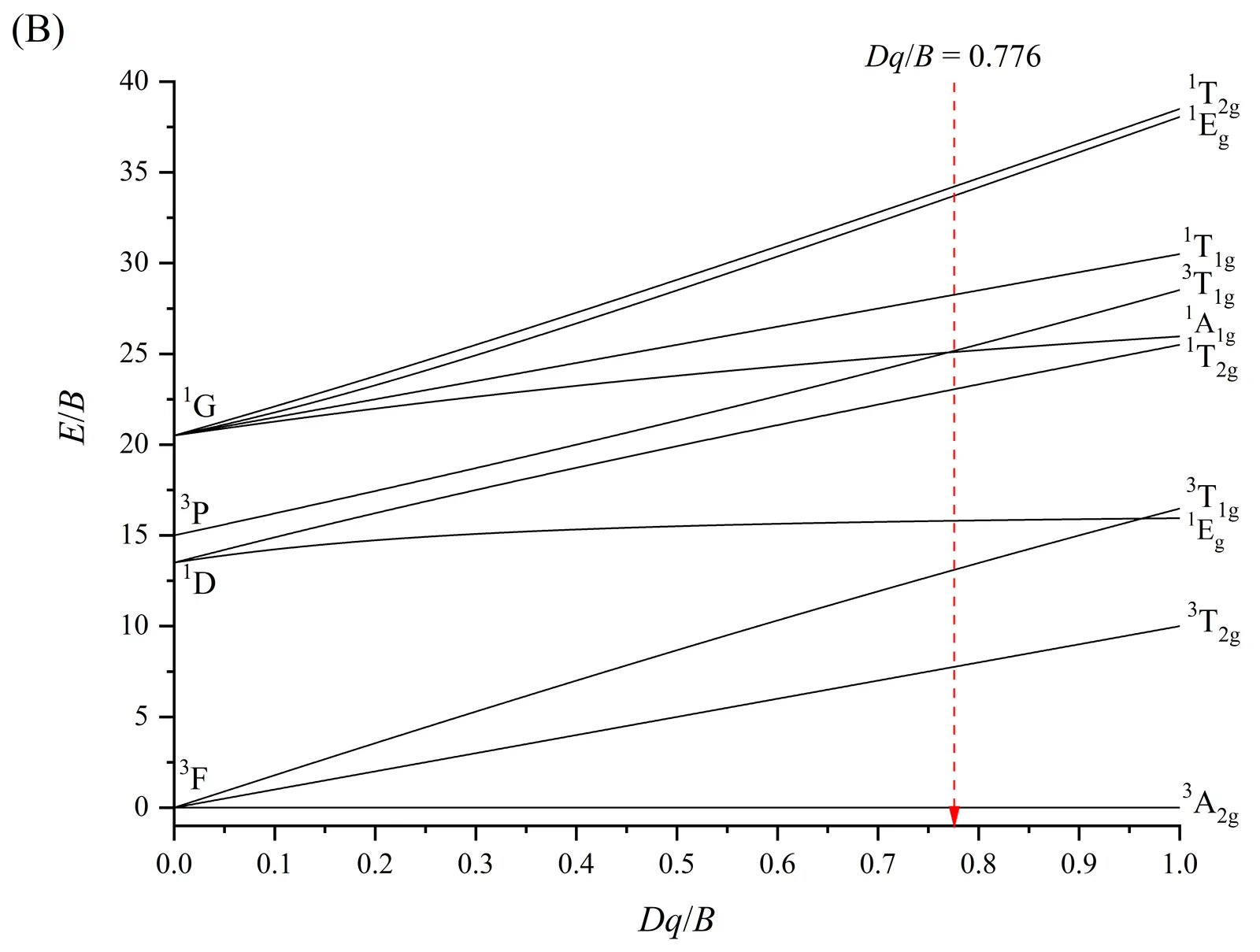
The Tanabe–Sugano diagram for Ni^2+^ ions in the Li_2_B_4_O_7_:Ni glass calculated for *C*/*B* = 4.25 and plotted in the wide (**A**) and narrow (**B**) ranges of the *Dq*/*B* values. The vertical position corresponding to the experimentally determined *Dq*/*B* = 0.776 is used to identify the relevant Ni^2+^ crystal-field states and transition energies.

The Tanabe–Sugano diagram for Ni^2+^ ions in the Li_2_B_4_O_7_:Ni glass plotted for *C*/*B* = 4.25 is shown in Figure 10. This diagram allows us to find theoretical *E*/*B* values (see Table 1) at the obtained above *Dq*/*B* = 0.776. The uncertainty of the *E*/*B* ratio was calculated individually for each state from the Tanabe–Sugano diagram plotted, taking into account the uncertainty of the *C*/*B* and *Dq*/*B* ratios. The dimensionless energies (*E*/*B*) of the relevant crystal-field states can be converted into absolute energies *E* and wavelengths *λ* using the value of *B*.

The calculated theoretical energies and wavelengths of all crystal field states of Ni^2+^ ions are listed in Table 1. The uncertainty of theoretical energies and wavelengths was calculated using the law of uncertainty propagation. Table 1 also presents the energies and wavelengths of Ni^2+^ transitions observed in the Li_2_B_4_O_7_:Ni glass. One can notice a quite good correlation between the observed and calculated wavelengths. Some deviations can be related to the distortion of octahedral geometry in the Li_2_B_4_O_7_:Ni glass.

The Ni^2+^ ions occupy the Li^+^ sites of the Li_2_B_4_O_7_ glass. The ionic radius of the B^3+^ ions is very small, being equal to 0.01 Å and 0.11 Å for three- and four-coordinated B^3+^ ions, respectively [32]. The ionic radii of Li^+^ and Ni^2+^ in the six-fold coordination are quite similar and equal approximately 0.76 Å and 0.69 Å [32], respectively, making substitution of Li^+^ by Ni^2+^ structurally plausible. The statement about the incorporation of Ni^2+^ ions into the Li^+^ well agrees with work [33], in which it was revealed by extended X-ray absorption fine structure (EXAFS) spectroscopy that rare-earth ions occupy the Li^+^ sites in the Li_2_B_4_O_7_ glass. The excess positive charges resulting from Ni^2+^ → Li^+^ heterovalent substitution in the investigated glass can be compensated by cationic vacancies as a characteristic feature of the glass network.

Based on the obtained results of optical spectroscopy, it is possible to state that the octahedral geometry around Ni^2+^ ions in the Li_2_B_4_O_7_:Ni glass is significantly distorted. In particular, the considerable spread of Li–O interatomic distances due to the positional disorder of the glass network manifests itself in the formation of distorted octahedra. This distortion is evident in the non-Gaussian shape with an additional weakly observed splitting of some observed optical absorption and emission bands, as well as the non-single-exponential luminescence decay kinetics of the Ni^2+^ emission.

The comparison of the experimentally observed wavelengths of the spin-allowed Ni^2+^ transitions in the optical absorption spectrum, the calculated Racah parameters, and the crystal field strength in the investigated Li_2_B_4_O_7_:Ni glass and materials with similar composition is presented in Table 2. The values obtained in the present study are in good agreement with those reported previously for Ni-doped borate glasses [15,16,17,18,34,35,36,37]. It should be noted that Refs. [15,16,17,18,35] report only the results of optical absorption without the analysis of crystal field splitting or determination of the corresponding Racah parameters, which underlines the significance of this work. Hence, the results of this comprehensive study provide valuable information about the structural, optical, and luminescent properties as well as the crystal field analysis of the Ni-doped Li_2_B_4_O_7_ glass.

## 4. Conclusions

Based on the analysis of the obtained experimental results, the following conclusions can be proposed:

• The Li_2_B_4_O_7_ (Li_2_O–2B_2_O_3_) glass doped with 1.0 mol.% NiO was successfully synthesised by the conventional melt-quenching method and has been studied in detail using XRD, optical absorption, and photoluminescence (emission and excitation spectra, decay kinetics) techniques.
• The absence of sharp crystalline diffraction peaks in XRD pattern confirms the disordered glassy (vitreous) structure of the investigated material.
• The optical absorption spectrum exhibits three broad bands at 428 nm, 815 nm, and 1417 nm corresponding to the spin-allowed ^3^A_2g_ (^3^F) → ^3^T_2g_ (^3^F), ^3^A_2g_ (^3^F) → ^3^T_1g_ (^3^F), and ^3^A_2g_ (^3^F) → ^3^T_1g_ (^3^P) transitions of Ni^2+^ (3*d*^8^) ions in the ligand field of octahedral geometry.
• In the luminescence spectra, the green emission band with a maximum at 525 nm was assigned to the ^3^T_1g_ (^3^P) → ^3^A_2g_ (^3^F) transitions with possible contributions from nearby spin-forbidden ^1^A_1g_ (^1^G) → ^3^A_2g_ (^3^F), and ^1^T_2g_ (^1^D) → ^3^A_2g_ (^3^F) transitions, whereas the red emission band with a maximum at 687 nm (14,556 cm^−1^) was ascribed to the ^1^E_g_ (^1^D) → ^3^A_2g_ (^3^F) transition of Ni^2+^ ions. Additionally, two weak emission bands related to the ^3^T_1g_ (^3^F) → ^3^A_2g_ (^3^F) and ^3^T_2g_ (^3^F) → ^3^A_2g_ (^3^F) transitions were observed in the spectral ranges of 900–1100 nm and 1500–1750 nm, respectively.
• The excitation spectra of the Li_2_B_4_O_7_:Ni glass reveal several spin-allowed and spin-forbidden 3*d*–3*d* transitions of Ni^2+^ ions.
• The luminescence decay curves of the green and red emission bands are non-single-exponential and are characterised by lifetimes distributed over a relatively wide time range, which is caused by the superposition of Ni^2+^ transitions, local symmetry lowering, as well as the positional disorder of the Li_2_B_4_O_7_:Ni glass network.
• The octahedral geometry around Ni^2+^ ions in the Li_2_B_4_O_7_:Ni glass is distorted due to the positional disorder of the glass network. The non-Gaussian shape of some observed optical absorption and emission bands, as well as deviations between the observed and calculated energies of some transitions, are related to the distorted octahedral ligand field.
• Analysis of the crystal field splitting for Ni^2+^ ions in the Li_2_B_4_O_7_:Ni glass was carried out, and the Racah parameters and the crystal field strength were calculated to be *B* = 929 ± 10 cm^−1^, *C* = 3950 ± 170 cm^−1^, and *Dq* = 720 ± 15 cm^−1^.
• The Tanabe–Sugano diagram for Ni^2+^ ions in the Li_2_B_4_O_7_:Ni glass was built, and quite good agreement between the observed and calculated wavelengths of the Ni^2+^ transitions was obtained.
• Overall, the valuable information gathered in this research is useful for understanding the structural and optical properties of Ni^2+^ ions in borate glasses.

## Figures and Tables

**Table 1:** The observed wavelength (*λ*_obs_) and energy (*E*_obs_) of the transitions between the ground and excited states, as well as the calculated relative energy (*E*/*B*), absolute energy (*E*_cal_), and wavelength position (*λ*_cal_) of the excited Ni^2+^ crystal field states in the Li_2_B_4_O_7_:Ni glass evaluated using the Tanabe–Sugano model with *Dq*/*B* = 0.776 and *C*/*B* = 4.25. The superscripts ^(abs)^, ^(exc)^, and ^(em)^ denote transitions observed in the absorption, excitation, and emission spectra, respectively.

Crystal Field State	λ_obs_ [nm]	E_obs_ [cm^−1^]	E/B	E_cal_ [cm^−1^]	λ_cal_ [nm]
^1^A_1g_ (^1^S)	—	—	62.67 ± 1.94	58,200 ± 2420	172 ± 7
^1^T_2g_ (^1^G)	296 ± 1 ^(exc)^	33,784 ± 114 ^(exc)^	34.22 ± 0.92	31,780 ± 1190	315 ± 12
^1^E_g_ (^1^G)	—	—	33.72 ± 0.92	31,310 ± 1190	319 ± 12
^1^T_1g_ (^1^G)	381 ± 1 ^(exc)^	26,247 ± 69 ^(exc)^	28.26 ± 0.70	26,240 ± 930	381 ± 13
^3^T_1g_ (^3^P)	428 ± 1 ^(abs)^	23,364 ± 55 ^(abs)^	25.17 ± 0.54	23,380 ± 750	428 ± 14
^1^A_1g_ (^1^G)	—	—	25.10 ± 0.42	23,310 ± 640	429 ± 12
^1^T_2g_ (^1^D)	467 ± 1 ^(exc)^	21,413 ± 46 ^(exc)^	23.06 ± 0.73	21,410 ± 900	467 ± 20
^1^E_g_ (^1^D)	687 ± 1 ^(em)^	14,556 ± 21 ^(em)^	15.80 ± 0.48	14,680 ± 600	681 ± 28
^3^T_1g_ (^3^F)	815 ± 1 ^(abs)^	12,270 ± 15 ^(abs)^	13.11 ± 0.37	12,170 ± 470	821 ± 32
^3^T_2g_ (^3^F)	1417 ± 1 ^(abs)^	7057 ± 5 ^(abs)^	7.76 ± 0.24	7210 ± 300	1388 ± 58

**Table 2:** The comparison of the observed wavelengths (λ_1_, λ_2_, λ_3_) of the ^3^A_2g_ (^3^F) → ^3^T_1g_ (^3^P), ^3^A_2g_ (^3^F) → ^3^T_1g_ (^3^F), and ^3^A_2g_ (^3^F) → ^3^T_2g_ (^3^F) spin-allowed transitions in the optical absorption spectrum, the calculated Racah parameters (*B* and *C*) and the crystal field strength (*Dq*) determined for the Li_2_B_4_O_7_:Ni glass and reported for selected Ni-doped materials. A dash (—) indicates that the corresponding parameter was not reported or could not be determined from the cited study.

Composition	λ_1_ [nm]	λ_2_ [nm]	λ_3_ [nm]	*B* [cm^−1^]	*C* [cm^−1^]	*Dq* [cm^−1^]	Reference
Li_2_B_4_O_7_:NiO(1 mol.%) or 33Li_2_O–66B_2_O_3_–1NiO glass	428 ± 1	815 ± 1	1417 ± 1	929 ± 10	3950 ± 170	720 ± 15	this work
19.537Li_2_O–80B_2_O_3_–0.463NiO glass	416	—	—	—	—	—	[15]
20Na_2_O–15ZnO–64B_2_O_3_–1NiO glass	424	799	—	920	—	743	[16]
29Bi_2_O_3_–70B_2_O_3_–1NiO glass	423	—	—	—	—	—	[17]
49.5PbO–50B_2_O_3_–0.5NiO glass	425	793	1264	—	—	—	[18]
LiF–B_2_O_3_ glass with 1 mol.% Ni(II)	440	840	1471	925	3790	680	[34]
30Li_2_O–10BaO–59.6B_2_O_3_–0.4NiO glass	443	810	1310	—	—	—	[35]
69.5B_2_O_3_–25Na_2_O–5PbO–0.5NiO glass	428	808	—	912	—	715.5	[36]
β-BaB_2_O_4_:Ni(6.86wt.%) nanopowder	400	685	1100	850	3500	900	[37]

## Data Availability

The original contributions presented in this study are included in the article. Further inquiries can be directed to the corresponding author.
